# Curcumin Inhibits Hepatocellular Carcinoma via Regulating miR-21/TIMP3 Axis

**DOI:** 10.1155/2020/2892917

**Published:** 2020-07-17

**Authors:** Jingtao Li, Hailiang Wei, Yonggang Liu, Qian Li, Hui Guo, Yingjun Guo, Zhanjie Chang

**Affiliations:** ^1^Department of Liver Diseases, The Affiliated Hospital of Shaanxi University of Chinese Medicine, Xianyang 712046, Shaanxi, China; ^2^Department of General Surgery, The Affiliated Hospital of Shaanxi University of Chinese Medicine, Xianyang 712046, Shaanxi, China; ^3^Medical Experiment Center, The Shaanxi University of Chinese Medicine, Xianyang 712046, Shaanxi, China; ^4^Department of Infectious Diseases, Ningxia People's Hospital, Yinchuan 750001, Ningxia, China

## Abstract

**Background/Aim:**

Curcumin exhibits anticancer effects against various types of cancer including hepatocellular carcinoma (HCC). miR-21 has been reported to be involved in the malignant biological properties of HCC. However, whether miR-21 plays a role in curcumin-mediated treatment of HCC is unknown. The purpose of this study was to identify the potential functions and mechanisms of miR-21 in curcumin-mediated treatment of HCC.

**Methods:**

The anticancer effects of curcumin were assessed in vivo and in vitro. The underlying mechanism of miR-21 in curcumin-mediated treatment of HCC was assessed by quantitative real-time PCR (RT-qPCR), western blot, and Dual-Luciferase Reporter assays.

**Results:**

The present study revealed that curcumin suppressed HCC growth in vivo and inhibited HCC cell proliferation and induced cell apoptosis in a dose-dependent manner in vitro. Meanwhile, the curcumin treatment can downregulate miR-21 expression, upregulate TIMP3 expression, and inhibit the TGF-*β*1/smad3 signaling pathway. miR-21 inhibition enhanced the effect of curcumin on cell proliferation inhibition, apoptosis, and TGF-*β*1/smad3 signaling pathway inhibition in HepG2 and HCCLM3 cells. It demonstrated that TIMP3 was a direct target gene of miR-21. Interestingly, the effect of miR-21 inhibition on cell proliferation, apoptosis, and TGF-*β*1/smad3 signaling pathway in HepG2 and HCCLM3 cells exposed to curcumin was attenuated by TIMP3 silencing.

**Conclusion:**

Taken together, the present study suggests that miR-21 is involved in the anticancer activities of curcumin through targeting TIMP3, and the mechanism possibly refers to the inhibition of TGF-*β*1/smad3 signaling pathway.

## 1. Introduction

Hepatocellular carcinoma (HCC) is the most common primary liver cancer, ranking the fifth most prevalent cancer worldwide and the third most common cause of cancer-related death [1]. Several clinical protocols, including conventional chemotherapy, liver transplantation, surgical resection, and radiofrequency ablation, have been used to treat HCC [2]. However, these treatments are often ineffective due to late diagnosis, frequent recurrence, and low objective response rate [3], resulting in the 5-year overall survival rate of HCC patients remaining less than 18% [4]. Therefore, there is an urgent need to develop effective and safe therapeutic agents to improve the efficacy of HCC therapy.

Curcumin, a polyphenol extracted from the root and rhizome of turmeric (Curcuma Longa), exhibits strong antioxidant activities and anti-inflammatory and antitumor activities with low cytotoxicity to normal cells [5, 6]. Curcumin has been reported to inhibit the proliferation of various types of cancer, including HCC. Curcumin exerts powerful anticancer properties mainly by regulating a series of signaling pathways and molecular targets, such as PI3K/Akt, Wnt/*β*-catenin, TGF-*β*1/smad3, IGF, and VEGF [7–9]. Although a series of mechanisms have been proposed regarding curcumin as an antitumor, the exact molecular mechanism of curcumin against HCC is not fully understood.

It has been documented that circulating miR-21 is upregulated in HCC patients, which can be used as a diagnostic marker and therapeutic target for HCC and correlated with distant metastasis [10, 11]. miR-21 can promote the growth, migration, and invasion of HCC cells by targeting downstream target genes, such as FASLG, SOCS6, and KLF5 [12–14]. TIMP3, belonging to the TIMPs family, binds to the extracellular matrix and participates in the regulation of inflammation, cell proliferation, and migration. TIMP3 functions as a tumor suppressor in a variety of cancers, and its expression in liver cancer is silenced [15]. miR-21 has been reported to promote cell proliferation and invasion in renal and esophageal cancers by regulating TIMP3 [16, 17]. However, whether miR-21 can promote the malignant phenotypes of HCC by regulating TIMP3 has not been reported. Meanwhile, some researchers have implicated the miRNAs' interaction or interference with cancer drugs. Mudduluru et al. [18] revealed that curcumin inhibits invasion and metastasis of colorectal cancer by regulating miR-21 expression. These findings encouraged us to speculate that curcumin may exert cytotoxic effects against HCC cells by regulating the miR-21/TIMP3 axis. In this study, we investigated the role and possible mechanism of miR-21 in the antitumor effect of curcumin on HCC. The findings from this investigation provide the basis for the potential mechanism of curcumin against HCC and promote its clinical application in the treatment of HCC.

## 2. Materials and Methods

### 2.1. Animals

BALB/c nude mice (16–18 g, 4-5 weeks old) were purchased from the animal center of the West China Medical College of Sichuan University, Chengdu, China. All animals were kept under controlled conditions with 12 h light/12 h dark cycle and fed with free access to food and water. Animal care and experiments were performed in accordance with the National Institutes of Health Guide for the Care and Use of Laboratory Animals (NIH Publications no. 8023, revised 1978) and were approved by the Ethical Committee of The Affiliated Hospital of Shaanxi University of Chinese Medicine (Shaanxi, China).

### 2.2. Cell Culture

The human hepatoma cell lines HepG2 (CL-0103) and HCCLM3 (CL-0278) were obtained from Procell Life Science & Technology Co., Ltd. (Wuhan, China). All cells were cultured in Dulbecco's modified Eagle's medium (DMEM; HyClone, UT, USA) containing 10% fetal bovine serum (FBS; Gibco, CA, USA) and 1% penicillin-streptomycin (Beijing Solarbio Science & Technology Co., Ltd., Beijing, China) under a humidified incubator with 5% CO_2_ at 37°C.

### 2.3. Transfection

The miR-21 mimic, inhibitor, and negative control (NC) plasmids were purchased from Guangzhou RiboBio Co., Ltd. The siRNA against TIMP3 (si-TIMP3) and negative control siRNA (si-NC) were chemically synthesized by Shanghai GenePharma Technology Co., Ltd. All the in vitro transfection was performed using Lipofectamine® 2000 (Invitrogen, CA, USA) according to the manufacturer's protocol with 50 pmol/ml miR-21 mimic, inhibitor, and negative control, or 40 pmol/ml si-TIMP3 and si-NC. Before transfection, HepG2 or HCCLM3 cells were seeded into a 6-well plate at a density of 1 × 10^5^ cells/well and allowed to grow to 70–80% confluence. Transfection efficiency was determined using RT-qPCR assay following transfection for 24 h. The si-TIMP3 and si-NC sequences were shown as follows: si-TIMP3 sense strand: 5′-GCAUAAUCUGAGCCCUGCU-3′ and antisense strand, 5′-AGCAGGGCUCAGAUUAUGC-3′; si-NC sense strand 5′-UUCUCCGAACGUGUCACGUTT-3′ and antisense strand, 5′-ACGUGACACGUUCGGAGAATT-3′.

### 2.4. Cell Proliferation Assay

To evaluate cell viability, HepG2 or HCCLM3 cells (6 × 10^3^/well) were seeded in 96-well culture plates and incubated in an atmosphere containing 5% CO_2_ at 37°C. When the cells attained 60 or 70% confluence, cells were treated with different concentrations of curcumin and maintained for 24 h. To evaluate the effect of miR-21 or TIMP3 on cell viability, HepG2 and HCCLM3 cells were transfected with miR-21 inhibitor and/or si-TIMP3 sequence for 6 h and then treated with 40 *μ*M curcumin incubated for additional 24 h. Subsequently, 10 *μ*l CCK-8 diluted in fresh medium (100 *μ*l) was added to each well and incubated for an additional 1 h at 37 C. The absorbance of each well was measured at 450 nm using a microplate reader (Thermo Fisher Scientific, Inc.). All experiments were performed in triplicate.

### 2.5. Annexin-V/Propidium Iodide (PI) Double-Staining and Flow Cytometry Assay

To evaluate cell apoptosis, HepG2 or HCCLM3 cells (1 × 10^5^/well) were seeded in 6-well culture plates and incubated in an atmosphere containing 5% CO_2_ at 37°C. When the cells attained 60 or 70% confluence, cells were treated with different concentrations of curcumin and maintained for 24 h. To evaluate the effect of miR-21 or TIMP3 on cell apoptosis, HepG2 and HCCLM3 cells were transfected with miR-21 inhibitor and/or si-TIMP3 sequence for 6 h and then treated with 40 *μ*M curcumin incubated for additional 24 h. Following washing, trypsin digestion, and centrifugation, HepG2 or HCCLM3 cells were resuspended in 100 *μ*l binding buffer (1 × 10^5^ cells) with 5 *μ*l Annexin V-FITC and 5 *μ*l PI (BD Pharmingen, NJ, USA) for 20 min in the dark. Then, cell apoptosis was analyzed by a using a FACSCalibur™ Flow Cytometer (BD Biosciences) within 1 h.

### 2.6. RNA Extraction and RT-qPCR Analysis

TRIzol^®^ reagent (Takara Biotechnology, Co., Ltd.) was used to extract total RNA from the HepG2 and HCCLM3 cells according to the manufacturer's protocol. For detecting the TIMP3 mRNA expression, the reverse transcription (RT) reaction was carried out using PrimeScript™ RT reagent Kit (Takara Biotechnology, Co., Ltd.) and the qPCR was performed using SYBR^®^ Premix Ex Taq II (Takara). To quantify miRNA expression, RT reaction and qPCR were performed using Bulge-Loop™ miRNA RT-qPCR Primer and Bulge-Loop™ miRNA RT-qPCR Starter kit (Guangzhou RiboBio Co., Ltd.). The 2^−ΔΔCT^ value was used for comparative quantitation [19]. GAPDH and U6 small nuclear RNA genes were used as endogenous normalization controls. All primer sequences (Sangon Biotech Co., Ltd.) are presented in Table 1. The catalogue number of miR-21-5p primer is miRA0000076-1-100, and the catalogue number of U6 is miRAN0002-1-200.

### 2.7. Western Blot Assay

Total protein was isolated from treated HepG2 and HCCLM3 cells using RIPA lysis buffer containing protease inhibitors (Roche, Indianapolis, IN, USA). The protein concentrations across samples were measured using BCA protein assay kit (Beyotime, Shanghai, China). Equal amounts (20 *μ*g) of protein were separated by 10% SDS-PAGE and then transferred onto PVDF membranes (EMD Millipore). After blocking with 5% skim milk, the membranes were incubated at 4°C overnight with primary antibodies against TIMP3 (ab39184; 1 : 1,000), TGF-*β*1 (ab9785; 1 : 200), p-smad3 (ab63403; 1 : 500), cleaved caspase-3 (ab2302), Bax (ab53154), Bcl-2 (ab196495), and *β*-actin (ab8227; 1 : 2,000) all from Abcam (Cambridge, MA, UK), followed by HRP-conjugated goat anti-rabbit IgG (ab7090; 1 : 10,000) at room temperature for 1 h. Signals were detected using an ECL kit (Millipore). *β*-actin served as an endogenous reference.

### 2.8. Luciferase Reporter Assay

For luciferase reporter assay, the TIMP3 3′ untranslated region (UTR) encompassing the wild-type (wt) or mutant (mut) fragments of the miR-21 binding site was amplified by PCR and then cloned into a pmirGLO Dual-Luciferase miRNA Target Expression Vector (Promega, WI, USA) to form the reporter vector, namely, TIMP3 wt and TIMP3 mut, respectively. HepG2 and HCCLM3 cells were plated onto 24-well plates (5 × 10^4^ cells/well) and allowed to reach 50% confluence. Then, cells were cotransfected with recombinant plasmids (TIMP3 wt or TIMP3 mut), together with Renilla luciferase pRL-TK plasmid (Promega) and miR-21 mimic or scramble oligonucleotide using Lipofectamine^®^ 2000 reagent (Invitrogen; Thermo Fisher Scientific, Inc.). After transfection for 24 h, luciferase activities in each group were calculated using Dual-Luciferase Reporter Assay Kit (Promega) according to the manufacturer's instructions. Firefly luciferase activity was normalized to Renilla luciferase activity for each tested well.

### 2.9. Immunohistochemistry (IHC)

Tumor samples were fixed with 4% paraformaldehyde for 48 h, embedded in paraffin, and 5 *μ*m thick paraffin sections were prepared. Sections were deparaffinized, rehydrated, and subjected to antigen retrieval by 3% hydrogen peroxide. Then, the sections were incubated with the primary antibodies against TIMP3 (ab39184; Abcam, Cambridge, USA) overnight at 4˚C. Primary antibodies were detected using a biotin-conjugated goat anti-rabbit IgG (BA1003; Wuhan Boster Biological Technology, Ltd.) at 37˚C for 30 min. Following rinsing with PBS three times, the sections were exposed to 3,3′-diaminobenzidine (DAB; OriGene Technologies, Inc.) for 5 min and counterstained with hematoxylin. Brown or yellow granules in the cytoplasm or nucleus were considered positive immune staining. A Nikon imaging system was used for image collection.

### 2.10. Tumor Xenografts

200 *μ*l sterile PBS containing a HepG2 cell suspension (2 × 10^5^) was subcutaneously injected into BALB/c nude mice. Once the tumor diameter reached 4–6 mm, the mice were divided into 2 groups (*n* = 6): control and curcumin groups. Mice in the curcumin group were treated with 100 mg/kg curcumin daily for two weeks by intraperitoneal injection. Mice in the control group were injected with an equal volume of the solvent. At day 24, mice were sacrificed by CO_2_ inhalation, and tumors were removed and measured. Tumor volume was calculated as *T* (mm^3^) = length (mm) × width (mm^2^)/2.

### 2.11. Statistical Analysis

The statistical analysis was performed using SPSS 20.0 software (IBM Corp., Armonk, NY, USA). Values were presented as the mean ± standard deviation from three separate experiments. Differences among multiple groups were compared by one-way analysis of variance (ANOVA), and differences between two groups were compared by Student's *t*-test. *P* < 0.05 was considered statistically significant difference.

## 3. Results

### 3.1. Curcumin Suppresses HCC Growth in a Xenograft Model

**Figure 1:**
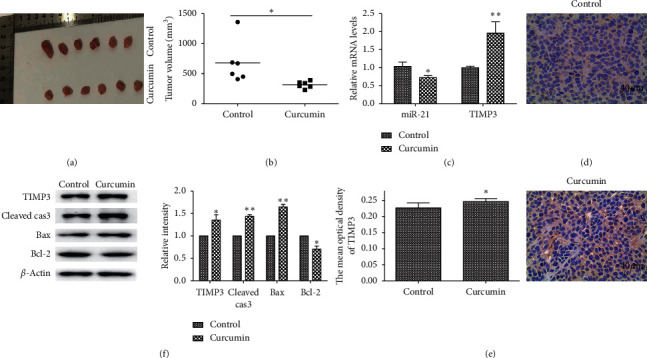
Effects of curcumin on tumor growth in a xenograft model. Tumors were harvested from a subset of HepG2 tumor mice on day 24 following cell injection. Ten days after inoculation, mice were treated with 100 mg/kg curcumin or an equal volume of the solvent daily for two weeks. (a) Images of tumors in the control and curcumin groups. (b) Tumor volume was measured at the end of the experiment. (c) The expression of miR-21 and TIMP3 mRNA was determined by RT-qPCR assay in vivo. (d, e) The expression of TIMP3 protein was determined by IHC assay in vivo. (f) The expression of TIMP3, cleaved caspase-3, Bax, and Bcl-2 proteins was determined by western blot assay in vivo. *n* = 6 per group. ^*∗*^*P* < 0.05 and ^*∗∗*^*P* < 0.01 vs. the control group.

In order to investigate the antitumor effects of curcumin in vivo, HepG2 cells were subcutaneously transplanted into the nude mice. As shown in Figures 1(a) and 1(b), curcumin administration has significantly suppressed tumor growth compared with control mice treated with solvent. Considering the fact that miRNAs play an important role in HCC, we first discussed the expression of miR-21 following curcumin treatment in vivo. The RT-qPCR results revealed that miR-21 expression was significantly downregulated (Figure 1(c)), indicating that miR-21 was important for the antitumor effect of curcumin. On the contrary, TIMP3 expression both in mRNA and protein levels was significantly increased in xenograft model of HCC (Figures 1(c)–1(f)). These data suggested that curcumin can regulate miR-21 and TIMP3 expression in HCC. In view of the negative regulatory association between miRNA and target genes, these data also suggested a potential correlation between miR-21 and TIMP3. In addition, the results also revealed that curcumin administration can significantly induce the expression of cleaved caspase-3 and Bax proteins and inhibit the expression of Bcl-2, indicating that the curcumin can induce HCC cell apoptosis in vivo (Figure 1(f)).

### 3.2. Curcumin Reduces Viability and Induces Apoptosis of HCC Cell Lines

**Figure 2:**
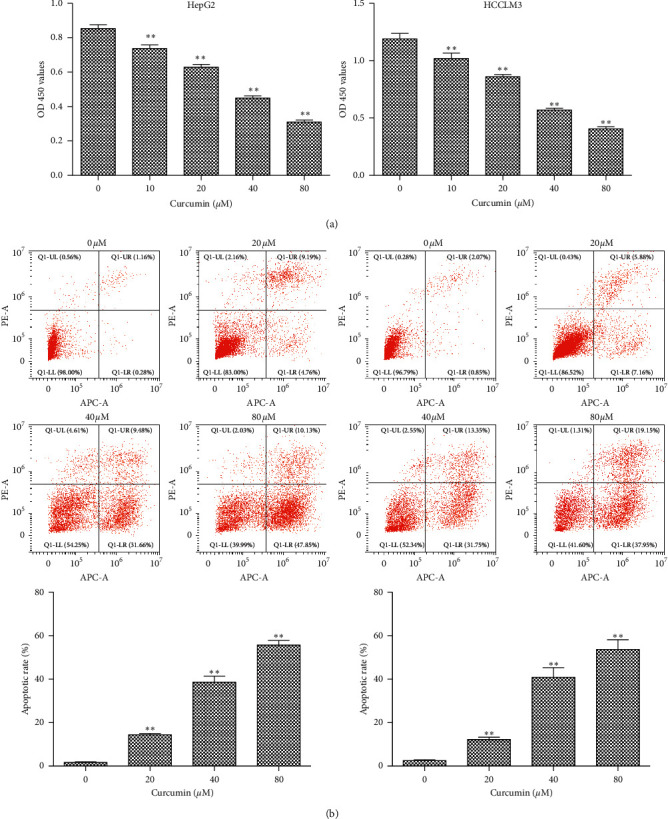
Effects of curcumin on the proliferation and apoptosis of HCC cell lines. HepG2 or HCCLM3 cells were treated with curcumin with indicated concentrations for 24 h. (a) Cell viability was evaluated by CCK-8 assay. (b) Apoptotic cells were detected by flow cytometry after staining with Annexin V-APC and Propidium Iodide. The experiment was repeated three times. Results are presented as the mean ± standard deviation. ^*∗∗*^*P* < 0.01 vs. the 0 *μ*M group.

In order to investigate the effects of curcumin on cell viability and apoptosis, HepG2 and HCCLM3 cells were treated with different concentrations of curcumin (0–80 *μ*M) for 24 h. As shown in Figure 2(a), both HepG2 and HCCLM3 cells showed a significant decrease in cell viability in a dose-dependent manner, with IC50 values of 30.45 ± 7.38 and 37.70 ± 6.37 *μ*M in HepG2 and HCCLM3 cells, respectively. Therefore, the concentration of 40 *μ*M was selected in the follow-up study. As shown in Figure 2(b), the flow cytometry results revealed that the cell apoptotic rate of HepG2 and HCCLM3 cells was significantly increased in a dose-dependent manner. These results indicated that curcumin can inhibit the proliferation and induce the apoptosis of HCC cells.

### 3.3. Curcumin Downregulates miR-21 Expression and Upregulates TIMP3 Expression in HCC Cell Lines

**Figure 3:**
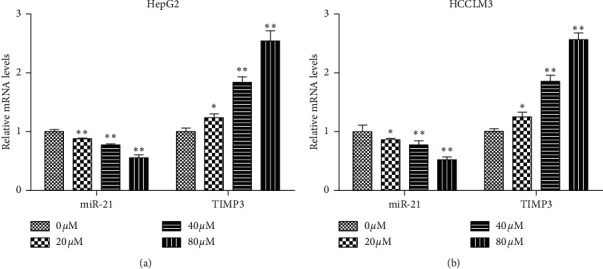
Effects of curcumin on the expression of miR-21 and TIMP3 in HCC cell lines. HepG2 or HCCLM3 cells were treated with curcumin with indicated concentrations for 24 h. The expression of miR-21 and TIMP3 mRNA was determined by RT-qPCR assay. The experiment was repeated three times. Results are presented as the mean ± standard deviation. ^*∗*^*P* < 0.05 and ^*∗∗*^*P* < 0.01 vs. the 0 *μ*M group.

The RT-qPCR results revealed that miR-21 expression was significantly downregulated in a dose-dependent manner in HCC cells (Figure 3). On the contrary, TIMP3 expression was significantly increased in a dose-dependent manner in HCC cells (Figure 3). These data suggested that curcumin can regulate miR-21 and TIMP3 expression in HCC cells, consistent with the results in vivo.

### 3.4. Curcumin Inhibits the TGF-*β*1/smad3 Signaling Pathway in HCC Cell Lines

**Figure 4:**
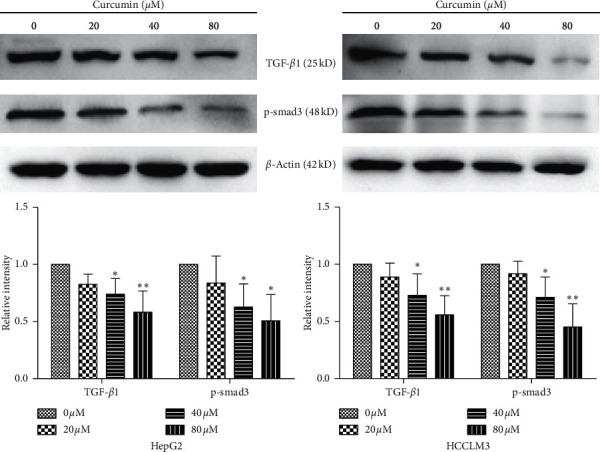
Effects of curcumin on TGF-*β*1/smad3 signaling pathway in HCC cell lines. HepG2 or HCCLM3 cells were treated with curcumin with indicated concentrations for 24 h. The expression of TGF-*β*1 and p-smad3 protein was determined by western blotting assay. The experiment was repeated three times. Results are presented as the mean ± standard deviation. ^*∗*^*P* < 0.05 and ^*∗∗*^*P* < 0.01 vs. the 0 *μ*M group.

Western blotting assay was applied to further investigate the potential signaling pathway associated with curcumin-regulated cell proliferation and apoptosis in HCC. The results demonstrated that curcumin treatment resulted in a downregulation in the expression of TGF-*β*1 and p-smad3 protein in a dose-dependent manner in HCC cells (Figure 4), indicating that the TGF-*β*1/smad3 signaling pathway was at least partially inhibited following curcumin treatment.

### 3.5. TIMP3 Is a Direct Target of miR-21 in HCC Cell Lines

**Figure 5:**
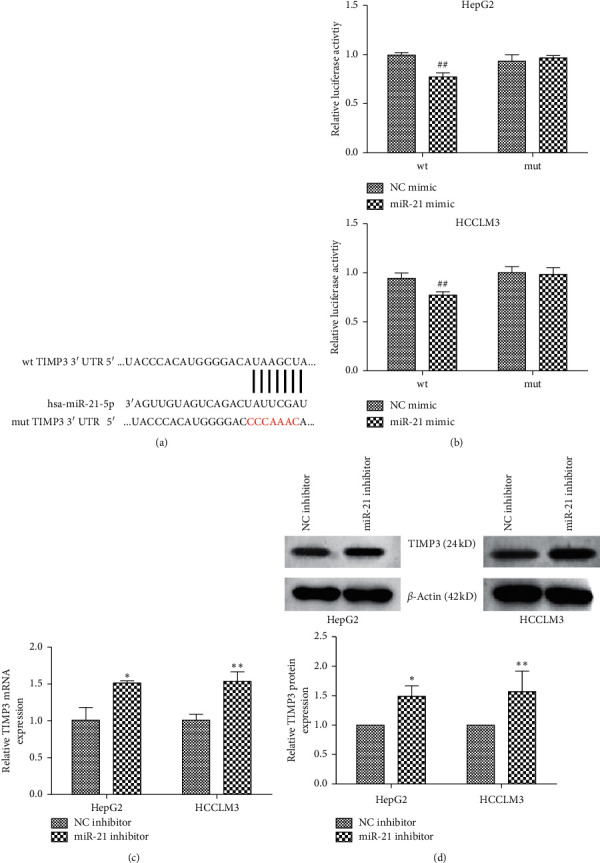
miR-21-5p targets TIMP3 in HCC cell lines. (a) The predicted miR-21-5p binding sites in the 3′-UTR of the TIMP3 gene. (b) The relative Luciferase activities were determined using a Dual-Luciferase Reporter Assay Kit following cotransfection with TIMP3-wt or TIMP3-mut and miR-21-5p mimic or NC mimic in HepG2 and HCCLM3 cells. (c) TIMP3 mRNA expression in HepG2 and HCCLM3 cells was measured by RT-qPCR assay following transfection with miR-21-5p inhibitor or NC inhibitor in HepG2 and HCCLM3 cells. (d) Western blotting was applied to quantify the expression level of TIMP3 following transfection with miR-21-5p inhibitor or NC inhibitor in HepG2 and HCCLM3 cells. The experiment was repeated three times; results were shown as means ± standard deviation. ^*∗*^*P* < 0.05 and ^*∗∗*^*P* < 0.01 vs. NC inhibitor group; ^##^*P* < 0.01 vs. NC mimic group.

Bioinformatics databases including TargetScan, miRDB, and StarBase were performed to predict the target of miR-21-5p. Among the predicted genes, TIMP3 was selected for further analysis. As a result, 3′-UTR of TIMP3 was contained in the complementary binding sequences for miR-21-5p (Figure 5(a)). To determine whether miR-21-5p directly binds to the 3′-UTR of TIMP3 mRNA, a luciferase reporter assay was performed. As shown in Figure 5(b), the luciferase activity of the wild type of TIMP3 3′-UTR was markedly decreased by miR-21-5p mimic in HepG2 and HCCLM3 cells, while the luciferase activity of the mutant type of TIMP3 3′-UTR has no obvious change. Meanwhile, the expression of TIMP3 mRNA and protein increased significantly following transfection with miR-21-5p inhibitor in HepG2 and HCCLM3 cells, respectively (Figures 5(c) and 5(d)). These data suggested that miR-21-5p targets TIMP3 and suppresses its expression in HCC cell lines.

### 3.6. Inhibition of miR-21 Enhances the Effect of Curcumin on Cell Proliferation Inhibition and Apoptosis in HCC Cell Lines

**Figure 6:**
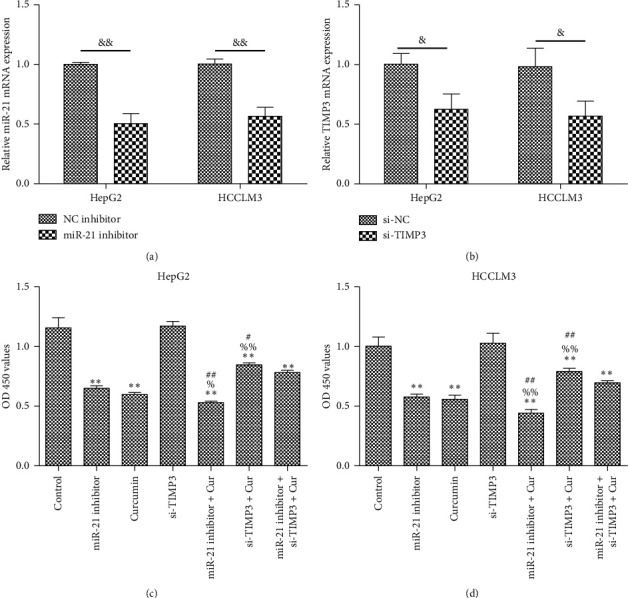
Effects of miR-21 inhibition and/or TIMP3 silencing on cell proliferation in HCC cell lines exposed to curcumin. (a, b) HepG2 and HCCLM3 cells were transfected with miR-21 inhibitor or si-TIMP3 sequence for 24 h. RT-qPCR was applied to quantify the expression of miR-21 and TIMP3. ^&^*P* < 0.05 and ^&&^*P* < 0.01. (c, d) HepG2 and HCCLM3 cells were transfected with miR-21 inhibitor and/or si-TIMP3 sequence for 6 h and then treated with 40 *μ*M curcumin incubated for additional 24 h. Cell viability was evaluated by CCK-8 assay. The experiment was repeated three times; results were shown as means ± standard deviation. ^*∗∗*^*P* < 0.01 vs. control group; ^%^*P* < 0.05 and ^%%^*P* < 0.01 vs. curcumin group; and ^#^*P* < 0.05 and ^##^*P* < 0.01 vs. miR-21 inhibitor+ si-TIMP3+ Cur group.

**Figure 7:**
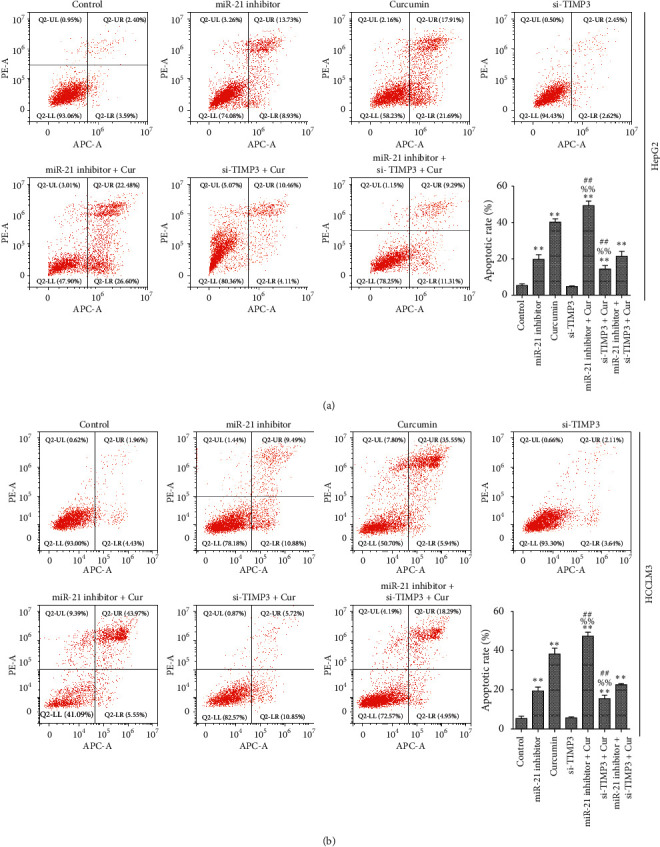
Effects of miR-21 inhibition and/or TIMP3 silencing on cell apoptosis in HCC cell lines exposed to curcumin. HepG2 and HCCLM3 cells were transfected with miR-21 inhibitor and/or si-TIMP3 sequence for 6 h and then treated with 40 *μ*M curcumin incubated for additional 24 h. (a, b) Apoptotic cells were detected by flow cytometry after stained with Annexin V-APC and Propidium Iodide in HepG2 and HCCLM3 cells, respectively. The experiment was repeated three times; results were shown as means ± standard deviation. ^*∗∗*^*P* < 0.01 vs. control group; ^%%^*P* < 0.01 vs. curcumin group; and ^##^*P* < 0.01 vs. miR-21 inhibitor+ si-TIMP3+ Cur group.

To determine the biological effects of miR-21 or TIMP3 in HCC cell lines exposed to curcumin, the HepG2 or HCCLM3 cells were transfected with miR-21 inhibitor or si-TIMP3 sequence and then treated with curcumin. RT-qPCR results revealed that the expression of miR-21 or TIMP3 was significantly decreased following transfection with miR-21 inhibitor or si-TIMP3 sequence, respectively (Figures 6(a) and 6(b)). These data indicated high transfection efficiency in HepG2 or HCCLM3 cells. Furthermore, cell proliferation and apoptosis were detected after transfection. The results showed that the proliferation of HepG2 and HCCLM3 cells was significantly decreased (Figures 6(c) and 6(d), while the apoptosis of HepG2 and HCCLM3 cells was significantly increased (Figures 7(a) and 7(b)) after transfection with miR-21 inhibitor or treatment with curcumin, while the proliferation and apoptosis of HepG2 and HCCLM3 cells have no obvious change after transfection with si-TIMP3 sequence, indicating that the inhibition of miR-21 may have a synergistic effect with curcumin on cell proliferation and apoptosis. Compared with curcumin group, the proliferation of HepG2 and HCCLM3 cells in miR-21 inhibitor+ Cur group was significantly decreased, while the proliferation in si-TIMP3+ Cur group was significantly increased, indicating that the inhibition of miR-21 enhanced the suppressive effect of curcumin on HepG2 and HCCLM3 cell proliferation, while the silencing of TIMP3 attenuated the suppressive effect of curcumin on cell proliferation. Compared with miR-21 inhibitor+ si-TIMP3+ Cur group, the proliferation of HepG2 and HCCLM3 cells in miR-21 inhibitor+ Cur group was significantly decreased, while the proliferation in si-TIMP3+ Cur group was significantly increased, indicating that the silencing of TIMP3 attenuated the suppressive effect of curcumin and miR-21 inhibitor on cell proliferation. Apoptosis of HepG2 and HCCLM3 cells showed an opposite trend compared with cell proliferation.

### 3.7. Inhibition of miR-21 Enhances the Inhibitory Effect of Curcumin on TGF-*β*1/smad3 Signaling Pathway in HCC Cell Lines

**Figure 8:**
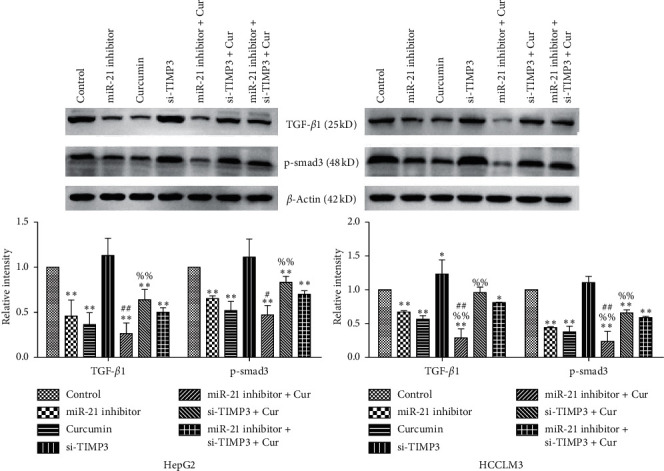
Effects of miR-21 inhibition and/or TIMP3 silencing on TGF-*β*1/smad3 signaling pathway inhibition induced by curcumin in HCC cell lines. HepG2 and HCCLM3 cells were transfected with miR-21 inhibitor and/or si-TIMP3 sequence for 6  h and then treated with 40 *μ*M curcumin incubated for additional 24 h. The expression of TGF-*β*1 and p-smad3 was evaluated by western blotting. The experiment was repeated three times; results were shown as means ± standard deviation. ^*∗*^*P* < 0.05 and ^*∗∗*^*P* < 0.01 vs. control group; ^%^*P* < 0.05 and ^%%^*P* < 0.01 vs. curcumin group; and ^#^*P* < 0.05 and ^##^*P* < 0.01 vs. miR-21 inhibitor+ si-TIMP3+ Cur group.

Western blotting assay was applied to further investigate the potential signaling pathway associated with miR-21-regulated cell proliferation and apoptosis in HCC. The results demonstrated that the expression of TGF-*β*1 and p-smad3 was significantly decreased after transfection with miR-21 inhibitor or treatment with curcumin, indicating that the inhibition of miR-21 may have a synergistic effect with curcumin on TGF-*β*1/smad3 signaling pathway inhibition. Compared with curcumin group, TGF-*β*1 and p-smad3 expression of HCCLM3 cells in miR-21 inhibitor+ Cur group was significantly decreased, while TGF-*β*1 and p-smad3 expression of HepG2 and HCCLM3 cells in si-TIMP3+ Cur group was significantly increased, indicating that the inhibition of miR-21 enhanced the suppressive effect of curcumin on TGF-*β*1/smad3 signaling pathway, while the silencing of TIMP3 attenuated the suppressive effect. Compared with miR-21 inhibitor+ si-TIMP3+ Cur group, TGF-*β*1 and p-smad3 expression of HepG2 and HCCLM3 cells in miR-21 inhibitor+ Cur group was significantly decreased, indicating that the silencing of TIMP3 attenuated the suppressive effect of curcumin and miR-21 inhibitor on TGF-*β*1/smad3 signaling pathway (Figure 8).

## 4. Discussion

HCC remains one of the most common malignant tumors in the digestive tract, despite the significant progress in the diagnosis and treatment. Recent studies have shown that curcumin plays an important role in inhibiting the progression of HCC through various pathways. However, the specific mechanisms of its inhibition of HCC are still uncertain. In the present study, we focused on the abnormal expression of miR-21 and investigated the regulation of miR-21 on the proliferation and apoptosis of curcumin-treated HCC cells via TIMP3/TGF-*β*1/smad3 signaling pathway.

Curcumin, the main active ingredient of turmeric, is widely used in Ayurvedic medicine in India and South Asia because it is nontoxic to normal cells and exhibits several beneficial properties [20]. In recent years, curcumin has attracted extensive attention because of its antitumor effect. Hu et al. [21] reported that curcumin inhibits breast cancer metastasis by inhibiting stem cell-like properties and EMT. Li et al. [22] showed that curcumin attenuates hyperglycemia-driven EGF-induced invasive and migratory abilities of pancreatic cancer via inhibiting the ERK and AKT signaling pathways. The antitumor effect of curcumin has also been observed in HCC. You et al. [23] reported that curcumin suppresses the growth of HCC via downregulating SREBF1. Curcumin also can decrease the proliferation activity of HCC SMMC-7721 cells via regulating AMPK signaling pathway [24]. In the present study, it was demonstrated that curcumin significantly inhibited HCC growth and induced HCC cell apoptosis in vivo and in vitro, suggesting that induced apoptosis may be an underlying mechanism for curcumin-mediated growth inhibition.

MicroRNAs (miRNAs), a class of small noncoding RNAs with a length of 19–24 nucleotides, negatively regulate gene expression by binding to the 3′-UTR of target mRNA to accelerate mRNA turnover or translational repression at the posttranscriptional level [25]. miRNAs are involved in fundamental cellular processes such as cell differentiation, proliferation, migration, and metabolism [26]. Indeed, emerging evidences indicate that miRNAs are closely linked to the antitumor capacity of curcumin. Li et al. [27] revealed an involvement of miR-99a/JAK/STAT signaling pathway in curcumin-induced proliferation, migration, and invasion inhibition of retinoblastoma. Li et al. [28] reported that curcumin inhibited proliferation and induced apoptosis of colorectal cancer via regulating the miR-491/PEG10 signaling pathway. miRNAs are useful biomarkers for diagnosis and prognosis of HCC, and the restoration of abnormally expressed miRNAs is considered to be an effective treatment for HCC [29, 30]. However, the interaction between curcumin and HCC related-miRNAs is rarely reported. Accumulating evidence indicates that miR-21 acts as a prosurvival factor in tumor cells [31, 32]. miR-21 can promote cell migration and invasion of HCC by targeting KLF5 [12]. Overexpression of miR-21 has promoted the recurrence in patients with hepatitis B virus-mediated HCC undergoing liver transplantation [33]. These studies suggest that miR-21 is an important therapeutic target against HCC. In the present study, it revealed that curcumin treatment has significantly decreased the expression of miR-21 in HCC cells. Inhibition of miR-21 enhanced the antiproliferative and proapoptotic effects of curcumin on HCC cells. These data indicated that the downregulation of miR-21 may be a novel mechanism for the anticancer activity of curcumin.

miRNAs regulation on tumors is realized by miRNA acting on specific target genes and signal transduction pathways. Endogenous inhibitors of matrix metalloproteinases (MMPs) play an important role in extracellular matrix (ECM) homeostasis and deregulate ECM remodeling which contributes to cancer growth, migration, and invasion, while TIMP3 can inhibit the function of MPPs. Several studies have proved that TIMP3 functions as a tumor suppressor in many malignant tumors, including HCC [34–36]. Wang et al. [37] reported that miR-181b inhibition negatively regulates the expression of TIMP3, inhibiting the occurrence of liver cancer. Notably, we found that curcumin treatment increased the expression of TIMP3 in HCC cells, indicating that the upregulation of TIMP3 may be a novel mechanism for the anticancer activity of curcumin. However, the relationship between miR-21 and TIMP3 in HCC remains unclear. In the present study, we found that TIMP3 is a direct target of miR-21 in HCC cells which was supported by data from the Luciferase assay, RT-qPCR, and western blotting. Interestingly, the silencing of TIMP3 attenuates the effect of miR-21 inhibition on curcumin-mediated cytotoxicity. These data indicated that the inhibition of miR-21 enhances the effect of curcumin on cell proliferation inhibition and apoptosis in HCC cells by targeting TIMP3.

The TGF-*β*1/smad3 signaling pathway, a critical regulator of apoptosis, mediates important biological processes including tumorigenesis [38]. Yu et al. [39] reported that genistein inhibits mouse colon cancer via regulating TGF-*β*1/smad pathway. Jiang et al. [40] demonstrated that miR-491 involved in arsenic trioxide-induced antiangiogenesis in HCC via inhibiting the TGF-*β*/smad3/NF-*κ*B signaling pathway. In the present study, it was revealed that curcumin treatment inhibited the expression of TGF-*β*1/smad3 signaling pathway-related proteins, indicating that the curcumin inhibited the malignant phenotype of HCC partially by inhibiting TGF-*β*1/smad3 signaling pathway. In addition, miR-21 inhibition enhanced the suppressive effect of curcumin on TGF-*β*1/smad3 signaling pathway, while the silencing of TIMP3 has attenuated the effect of miR-21 inhibition, indicating that the inhibition of miR-21 enhances the effect of curcumin on cell proliferation inhibition and apoptosis in HCC cells by regulating TIMP3/TGF-*β*1/smad3 axis.

## 5. Conclusion

In summary, the present study demonstrated that curcumin exhibits antiproliferative and proapoptotic activities in HCC, which are largely mediated through miR-21/TIMP3/TGF-*β*1/smad3 axis. These findings extend our understanding of the anticancer mechanism of curcumin and provide new targets for the treatment of HCC.

## Figures and Tables

**Table 1:** Primer sequences for RT-qPCR.

Gene	Primer sequence (5′⟶3′)
TIMP3	F: CGATGAGGTAATGCGGCTCT R: CATCTTGGTGAAGCCTCGGT
U6	F: GCTTCGGCAGCACATATACTAAAAT R: CGCTTCACGAATTTGCGTGTCAT
GAPDH	F: ACAGTCAGCCGCATCTTCT R: GACAAGCTTCCCGTTCTCAG

## Data Availability

The data used to support the findings of this study are available from the corresponding author upon request.

## References

[1] Chen W., Zheng R., Baade P. D. (2016). Cancer statistics in China, 2015. *CA: A Cancer Journal for Clinicians*.

[2] Forner A., Reig M. E., de Lope C. R., Bruix J. Current strategy for staging and treatment: the BCLC update and future prospects. *Seminars in Liver Disease*.

[3] Peters G. J., Honeywell R. J. (2015). Drug transport and metabolism of novel anticancer drugs. *Expert Opinion on Drug Metabolism & Toxicology*.

[4] Kulik L. M., Chokechanachaisakul A. (2015). Evaluation and management of hepatocellular carcinoma. *Clinics in Liver Disease*.

[5] Coban D., Milenkovic D., Chanet A. (2012). Dietary curcumin inhibits atherosclerosis by affecting the expression of genes involved in leukocyte adhesion and transendothelial migration. *Molecular Nutrition & Food Research*.

[6] Aggarwal B. B., Yuan W., Li S., Gupta S. C. (2013). Curcumin-free turmeric exhibits anti-inflammatory and anticancer activities: identification of novel components of turmeric. *Molecular Nutrition & Food Research*.

[7] Zhang L., Cheng X., Gao Y. (2016). Curcumin inhibits metastasis in human papillary thyroid carcinoma BCPAP cells via down-regulation of the TGF-*β*/Smad2/3 signaling pathway. *Experimental Cell Research*.

[8] Xu X. B., Qin J., Liu W. Curcumin inhibits the invasion of thyroid cancer cells via down-regulation of PI3K/Akt signaling pathway. *Gene*.

[9] Zheng R., Deng Q., Liu Y., Zhao P. Curcumin inhibits gastric carcinoma cell growth and induces apoptosis by suppressing the Wnt/*β*-catenin signaling pathway. *Medical Science Monitor International Medical Journal of Experimental & Clinical Research*.

[10] Zhang N., Hu Z., Qiang Y., Zhu X. (2019). Circulating miR-130b- and miR-21-based diagnostic markers and therapeutic targets for hepatocellular carcinoma. *Molecular Genetics & Genomic Medicine*.

[11] Guo X., Lv X., Lv X., Ma Y., Chen L., Chen Y. (2017). Circulating miR-21 serves as a serum biomarker for hepatocellular carcinoma and correlated with distant metastasis. *Oncotarget*.

[12] Wang J., Chu Y, Xu M, Zhang X, Zhou Y, Xu M (2019). miR-21 promotes cell migration and invasion of hepatocellular carcinoma by targeting KLF5. *Oncology Letters*.

[13] Li Z. B., Li Z-Z., Li L., Chu H.-T., Jia M. (2015). MiR-21 and miR-183 can simultaneously target SOCS6 and modulate growth and invasion of hepatocellular carcinoma (HCC) cells. *European Review for Medical and Pharmacological Sciences*.

[14] Chen S., Yang C., Sun C. (2019). miR-21-5p suppressed the sensitivity of hepatocellular carcinoma cells to cisplatin by targeting FASLG. *DNA and Cell Biology*.

[15] Casagrande V. Hepatocyte specific TIMP3 expression prevents diet dependent fatty liver disease and hepatocellular carcinoma. *Sci Rep*.

[16] Wang N. miR-21 down-regulation suppresses cell growth, invasion and induces cell apoptosis by targeting FASL, TIMP3, and RECK genes in esophageal carcinoma. *Digestive Diseases & Sciences*.

[17] Chen J., Gu Y., Shen W. (2017). MicroRNA-21 functions as an oncogene and promotes cell proliferation and invasion via TIMP3 in renal cancer. *European Review for Medical and Pharmacological Sciences*.

[18] Mudduluru G., George-William J. N., Muppala S. (2011). Curcumin regulates miR-21 expression and inhibits invasion and metastasis in colorectal cancer. *Bioscience Reports*.

[19] Livak K. J., Schmittgen T. D. (2001). Analysis of relative gene expression data using real-time quantitative PCR and the 2^−ΔΔCT^ method. *Methods*.

[20] Schraufstätter E., Bernt H. (1949). Antibacterial action of curcumin and related compounds. *Nature*.

[21] Hu C., Li M., Guo T. (2019). Anti-metastasis activity of curcumin against breast cancer via the inhibition of stem cell-like properties and EMT. *Phytomedicine*.

[22] Wang Y., Liang H., Jin F. (2019). Injured liver-released miRNA-122 elicits acute pulmonary inflammation via activating alveolar macrophage TLR7 signaling pathway. *Proceedings of the National Academy of Sciences of the United States of America*.

[23] You Z., Li B., Xu J., Chen L., Ye H. (2018). Curcumin suppress the growth of hepatocellular carcinoma via down-regulating SREBF1. *Oncology Research*.

[24] Zhang Y. J., Xiang H, Liu J. S, Li D, Fang Z. Y, Zhang H (2017). Study on the mechanism of AMPK signaling pathway and its effect on apoptosis of human hepatocellular carcinoma SMMC-7721 cells by curcumin. *European Review for Medical and Pharmacological Sciences*.

[25] Nilsen T. W. (2007). Mechanisms of microRNA-mediated gene regulation in animal cells. *Trends in Genetics*.

[26] Gregory R. I., Shiekhattar R. (2011). *MicroRNA Biogenesis and Cancer*.

[27] Li Y., Sun W., Han N., Zou Y., Yin D. (2018). Curcumin inhibits proliferation, migration, invasion and promotes apoptosis of retinoblastoma cell lines through modulation of miR-99a and JAK/STAT pathway. *BMC Cancer*.

[28] Li B., Shi C., Li B., Zhao J.-M., Wang L. (2018). The effects of Curcumin on HCT-116 cells proliferation and apoptosis via the miR-491/PEG10 pathway. *Journal of Cellular Biochemistry*.

[29] Jiang W., Zhang L., Guo Q. (2019). Identification of the pathogenic biomarkers for hepatocellular carcinoma based on RNA-seq analyses. *Pathology & Oncology Research*.

[30] Jin Y., Wong Y. S, Goh B. K. P (2019). Circulating microRNAs as potential diagnostic and prognostic biomarkers in hepatocellular carcinoma. *Scientific Reports*.

[31] Sun J., Jiang Z., Li Y., Wang K., Chen X., Liu G. (2019). Downregulation of miR-21 inhibits the malignant phenotype of pancreatic cancer cells by targeting VHL. *OncoTargets and Therapy*.

[32] Mo H., Guan J., Yuan Z.-C. (2019). Expression and predictive value of miR-489 and miR-21 in melanoma metastasis. *World Journal of Clinical Cases*.

[33] Dundar H. Z., Aksoy F., Aksoy S. A. (2019). Overexpression of miR-21 is associated with recurrence in patients with hepatitis B virus-mediated hepatocellular carcinoma undergoing liver transplantation. *Transplantation Proceedings*.

[34] Das A. M., Seynhaeve A. L. B., Rens J. A. P. (2014). Differential TIMP3 expression affects tumor progression and angiogenesis in melanomas through regulation of directionally persistent endothelial cell migration. *Angiogenesis*.

[35] Anania M. C., Sensi M., Radaelli E. TIMP3 regulates migration, invasion and in vivo tumorigenicity of thyroid tumor cells.

[36] Casagrande V., Mauriello A., Bischetti S., Mavilio M., Federici M., Menghini R. (2017). Hepatocyte specific TIMP3 expression prevents diet dependent fatty liver disease and hepatocellular carcinoma. *Scientific Reports*.

[37] Wang B., Hsu S.-H., Majumder S. (2010). TGF*β*-mediated upregulation of hepatic miR-181b promotes hepatocarcinogenesis by targeting TIMP3. *Oncogene*.

[38] Zhang T., Liang L., Liu X. TGF*β*1-Smad3-Jagged1-Notch1-Slug signaling pathway takes part in tumorigenesis and progress of tongue squamous cell carcinoma. *Journal of Oral Pathology & Medicine*.

[39] Yu Z., Tang Y., Hu D., Li J. (2005). Inhibitory effect of genistein on mouse colon cancer MC-26 cells involved TGF-*β*1/Smad pathway. *Biochemical and Biophysical Research Communications*.

[40] Jiang F., Wang X., Liu Q. Inhibition of TGF-*β*/SMAD3/NF-*κ*B signaling by microRNA-491 is involved in arsenic trioxide-induced anti-angiogenesis in hepatocellular carcinoma cells. *Toxicology Letters*.

